# Partial direct contact transmission in ferrets of a mallard H7N3 influenza virus with typical avian-like receptor specificity

**DOI:** 10.1186/1743-422X-6-126

**Published:** 2009-08-14

**Authors:** Haichen Song, Hongquan Wan, Yonas Araya, Daniel R Perez

**Affiliations:** 1 Department of Veterinary Medicine, University of Maryland, College Park and Virginia-Maryland Regional College of Veterinary Medicine, 8075 Greenmead Drive, College Park, MD 20742, USA; 2 Synbiotics Corporation, 8075 Greenmead Drive, College Park, MD 20742, USA; 3 Influenza Division, Centers for Disease Control and Prevention, 1600 Clifton Road, Atlanta GA 30333, USA

## Abstract

**Background:**

Avian influenza viruses of the H7 subtype have caused multiple outbreaks in domestic poultry and represent a significant threat to public health due to their propensity to occasionally transmit directly from birds to humans. In order to better understand the cross species transmission potential of H7 viruses in nature, we performed biological and molecular characterizations of an H7N3 virus isolated from mallards in Canada in 2001.

**Results:**

Sequence analysis that the HA gene of the mallard H7N3 virus shares 97% identity with the highly pathogenic avian influenza (HPAI) H7N3 virus isolated from a human case in British Columbia, Canada in 2004. The mallard H7N3 virus was able to replicate in quail and chickens, and transmitted efficiently in quail but not in chickens. Interestingly, although this virus showed preferential binding to analogs of avian-like receptors with sialic acid (SA) linked to galactose in an α2–3 linkage (SAα2–3Gal), it replicated to high titers in cultures of primary human airway epithelial (HAE) cells, comparable to an avian H9N2 influenza virus with human-like α2–6 linkage receptors (SAα2–6Gal). In addition, the virus replicated in mice and ferrets without prior adaptation and was able to transmit partially among ferrets.

**Conclusion:**

Our findings highlight the importance and need for systematic *in vitro *and *in vivo *analysis of avian influenza viruses isolated from the natural reservoir in order to define their zoonotic potential.

## Background

Influenza A viruses are classified based on the antigenic properties of the surface proteins hemagglutinin (HA) and neuraminidase (NA). To date, 16 HA and 9 NA subtypes have been described. Wild aquatic birds (Orders Anseriformes and Charadriiformes) are considered the major reservoir of influenza A viruses in nature [1]. In these birds, influenza viruses usually replicate in the intestinal tract, cause no disease, and spread by fecal contamination of water. These viruses occasionally infect terrestrial domestic birds (order Galliformes) and in a limited number of mammalian species including humans [2-5]. On rare occasions, these infections initiate horizontal chains of transmission and establishment of new host-adapted virus lineages.

Avian influenza viruses with the H7 HA (herein H7 viruses) have been associated with numerous outbreaks in poultry worldwide with devastating effects on the industry due to mass mortality, depopulation and trade restrictions [6]. Prior to 2003, infection with H7 viruses was not considered a serious health threat, although some H7 outbreaks in poultry or seals were sporadically associated with conjunctivitis by occupational exposure [7-9]. However, the H7N7 outbreak in the Netherlands in 2003 prompted a re-evaluation of the human health risks attributed to these viruses. The H7N7 virus infected at least 89 people with one case of fatal pneumonia. Although mild conjunctivitis was the most common presentation, mild influenza-like symptoms with respiratory involvement were also reported. Infections with H7 viruses may be more common than we currently recognize. Indeed, seroepidemiological studies revealed probable avian-to-human transmission of low pathogenic avian influenza (LPAI) H7 viruses in Italy [10]. In North America, avian H7 viruses have also been associated with human infections. Early in 2004, outbreaks of LPAI and HPAI H7N3 occurred among poultry in British Columbia, Canada [11]. Two poultry workers developed mild unilateral conjunctivitis, accompanied by coryza and headache. LPAI H7N3 was isolated from the respiratory secretions of one worker, whereas the HPAI H7N3 virus was isolated from conjunctival specimens from a second worker [12,13]. An isolated case of LPAI H7N2 virus infection was identified in an adult male from the New York metropolitan area, in November 2003, but the source of infection was not identified [14]. Thus, direct transmission of both HPAI and LPAI of the H7 subtype to humans highlight the need for a detailed molecular and biological characterization of H7 viruses *in vitro *and *in vivo *in order to better assess their zoonotic potential.

Recent studies show that some HPAI and LPAI H7 subtypes isolated from domestic poultry can infect mice and/or cause disease in ferrets without previous adaptation [15-17]. However little is known about the potential of H7 viruses isolated from wild aquatic birds to infect mammals. Understanding the potential of avian influenza viruses from the natural reservoir to infect various other animal species is crucial to determine their host range and zoonotic potential. Recently, Belser et al. [18] provided evidence that some contemporary Eurasian and North American influenza H7 viruses replicated efficiently in the upper respiratory tract of ferrets and were capable of direct contact transmission in this species.

In this study, we performed *in vitro *and *in vivo *characterizations of a A/mallard/Albetera/24/01 (H7N3) virus isolated in Alberta, Canada, 2001 (herein Mal/01). We found that the Mal/01 virus possesses characteristics of a typical avian influenza virus *in vitro*, i.e. it binds strongly to α2–3 avian-like receptors, and infects almost exclusively ciliated cells in air-liquid interface cultures of human airway epithelial cells. The Mal/01 virus transmitted to direct contact ferrets, although no airborne respiratory droplet transmission was observed. Our studies suggest that changes in receptor specificity, might not be necessarily required for some H7 viruses to infect and/or transmit in mammals. These studies highlight the importance of carefully scrutinizing the characteristics of viruses from the wild bird reservoir on a systematic basis in order to ascertain their zoonotic and pandemic potential.

## Results and discussion

### Sequence analysis of Mal/01 reveals close relationship to poultry H7 viruses that infected humans in British Columbia, Canada

The molecular features conducive to interspecies transmission of influenza viruses are largely unknown. We were interested in determining the biological and molecular characteristics as well as the host range of a typical mallard H7N3 virus that was circulating in wild ducks in Canada in 2001. This virus circulated in ducks in Canada prior to the H7 outbreak in poultry that transmitted to humans in 2004. Genome sequence analysis revealed that Mal/01 virus shares between 97.5% to 100% amino acid identity with the HPAI A/Canada/rv504/2004 (H7N3) virus with the exception of the NS gene (Table 1). While the NS gene of A/Canada/rv504/2004(H7N3) belongs to allele A, the Mal/01 H7N3 virus contains an NS gene that belongs to allele B. Mal/01 virus also shares more than 99.8% nucleotide sequence identity and more than 99.9% amino acid identity with another virus sequence deposited in Genbank, A/mallard/Alberta/34/2001 (H7N1) (Table 2). The N3 NA segment of Mal/01 is closely related to an H2N3 avian influenza virus, isolated from mallards in Canada in 2003 (Table 2). It must be noted that Mal/01 is a LPAI virus and in that regard is phenotypically different from the HPAI A/Canada/rv504/2004 (H7N3) virus. In addition to the different cleavage site, the HA of Mal/01 virus differs from the human isolate at three other positions as shown in Table 1. Taken together, these observations suggest that a potential precursor of the H7N3 virus that caused the outbreak in Canada was present in the wild duck population at least 3 years before the outbreak.

**Table 1:** Sequence comparison of Mal/01 versus A/Canada/rv504/2004(H7N3).

Virus gene segments (Gene accession NO.)	Identity, nt	Identity, aa	No. of substitution (aa)
PB2 (DQ017509.1)	94.7%	99.3%	5
PB1 (DQ017507.1)	97.7%	99.5%	4
PA (DQ017508.1)	98.7%	99.5%	3
HA (DQ017504.1)	97.0%	98.9%	3^a^
NP (DQ017514.1)	93.0%	98.8%	6
NA (DQ017503.1)	96.3%	97.5%	11
M (DQ017516.1)	97.5%	100%^b^	0^b^
NS (DQ017506.1)	67.4%	68.3%^c^	74^c^

^a ^Polybasic cleavage site in HA0 protein was excluded.

^b ^M1 protein

^c ^NS1 protein

**Table 2:** Influenza A viruses with greatest nucleotide and amino acid sequence identity to Mal/01 as determined by a BLAST search of the influenza virus database.

Virus gene segments	Identity, nt	Identity, aa	Virus designation
PB2	99.8%	99.9%	A/mallard/Alberta/34/2001(H7N1)
PB1	100%	100%	A/mallard/Alberta/34/2001(H7N1)
PA	99.9%	100%	A/mallard/Alberta/34/2001(H7N1)
HA	100%	100%	A/mallard/Alberta/34/2001(H7N1)
NP	99.8%	100%	A/mallard/Alberta/34/2001(H7N1)
NA	97.9%	99.1%	A/mallard/Alberta/79/2003(H2N3)
M	100%	100%^a^	A/mallard/Alberta/34/2001(H7N1)
NS	100%	100%^b^	A/mallard/Alberta/34/2001(H7N1)

^a^M1 protein identity

^b^NS1 protein identity

### *In vitro *characterization of RGMal/01 shows efficient replication in human airway epithelial cells despite strong α2–3 receptor specificity

**Figure 1:**
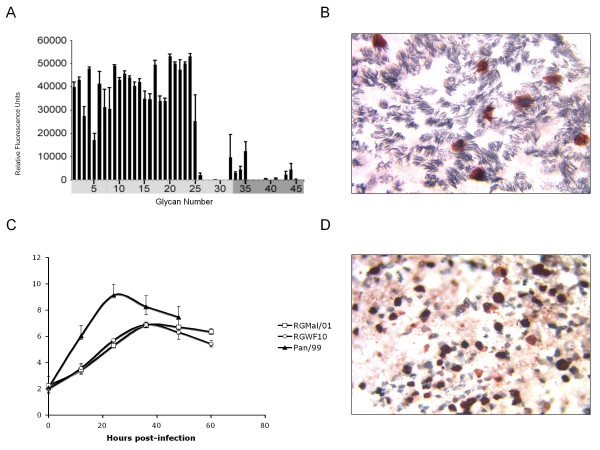
**Receptor specificity of RGMal/01 and single and multi round replication of the virus in HAE cells**. (A) Receptor specificity was measured by Glycan Array. Virus was analyzed at hemagglutination titers of 128 per 50 μl. The virus exhibited a strong preference for binding to mainly avian-type α 2–3 receptors, but not α 2–6 sialosides. 1–32 represent the glycans that contain α 2–3 SA (light gray), whereas 33–45 are the glycans with α 2–6 SA (dark gray). (B) HAE cultures were infected with RGMal/01 at an MOI of 1.0 and fixed at 7 hpi. The cilia (gray) and viral antigen (red) were visualized by double immunostaining. RGMal/01 is more efficient at infecting ciliated cells. (C) HAE cultures were inoculated via the apical side with the viruses at an MOI of 0.2. The progeny viruses released into the apical side were collected at the indicated time points and titrated in the MDCK cells by performing a TCID_50 _assay. Each bar represents the average for two independent experiments run with duplicate HAE cultures. (D) At end of the sampling (60 hpi) described from panel C, the cultures were fixed and stained for cilia (gray) and distribution of viral antigen (red).

We have previously shown that Mal/01 virus displayed exclusive preference for SAα2–3Gal-resialylated chicken red blood cells (CRBCs), whereas a prototypic human H3N2 virus (Pan/99) and an avian H9N2 virus (RGWF10) with human-like receptor specificity bound exclusively to SAα2–6Gal-resialylated CRBCs [19]. In this study, we further analyzed its receptor glycan preference using glycan microarray technology to survey more than 100 sialoglycans simultaneously [20]. Using reverse genetics we rescued the Mal/01 virus (RGMal/01) and generated a homogeneous population of virus corresponding to the most prevalent virus population in the original isolate. Analysis of the RGMal/01 virus with a microarray of sialoglycans revealed strong binding to multiple sialoside structures with α2–3 linkage to galactose(Fig 1A). Only minimal binding to a few α2–6 sialosides was detected (Fig 1A). These data demonstrated that the RGMal/01 virus is a typical avian influenza virus with nearly exclusive α2–3 sialoside receptor specificity.

Differentiated human airway epithelial (HAE) cell culture is a well-characterized *in vitro *model of human respiratory tract epithelium. The apical surface of HAE cells cultured at the air-liquid interface contains both ciliated cells and mucus-secreting non-ciliated cells. These cultures express SAα2–3Gal receptors predominantly on ciliated cells, while SAα2–6Gal receptors are presented mainly on non-ciliated cells [19,21-23]. Replication of influenza viruses in this *in vitro *model has defined a distinctive cell tropism pattern between avian and human influenza viruses based on their receptor preferences. While avian influenza viruses infect predominantly ciliated (SAα2–3Gal receptor) cells, human viruses replicate preferentially in non-ciliated cells (SAα2–6Gal receptors). Interestingly, avian influenza viruses that have acquired SAα2–6Gal receptor specificity show a human-like virus cell tropism by infecting mostly non-ciliated cells [19]. In addition, avian and human viruses that replicate in non-ciliated cells tend to produce more virus progeny compared to those that infect ciliated cells. Based on its preference binding to α2–3 sialosides and the origin of the Mal/01 virus, it was not unexpected to observe that it infected preferentially ciliated cells in HAE cultures within single round replication (Fig 1B). Thus, there was good agreement between the Mal/01 virus receptor specificity and its cell tropism in HAE cultures. We further examined the replication kinetics of RGMal/01 in HAE cultures. As controls, we included Pan/99 and RGWF10 which exhibit human-like receptor specificity and replicate preferentially in non-ciliated cells [19]. HAE cultures were inoculated on the apical side at a MOI of 0.2. Progeny viruses released into the apical were harvested and titrated in MDCK cells (Fig. 1C) whereas cell monolayers were fixed and stained to reveal cell type (cilia) and presence of viral antigen (Fig. 1D). The RGMal/01-infected HAE cells released ~100 times less virus than the human Pan/99 virus, but reached levels comparable to the avian RGWF10 H9N2 virus, which has human-like receptor binding specificity (Fig. 1C). The relatively efficient replication of the RGMal/01 virus in HAE cultures is supported by ciliated cells as indicated by the detection of viral antigen in these cells rather than in non-ciliated cells, even at 60 hpi (Fig. 1D). Our *in vitro* model results suggest that although the virus has high affinity for the avian-like α2–3-linked SA receptors, it may have the potential to replicate efficiently in epithelial cells of the human respiratory tract. Therefore, we analyzed the replication of RGMal/01 in avian and mammalian *in vivo* models.

### Replication and transmission of RGMal/01 in domestic land-based birds

To determine whether RGMal/01 could readily replicate and transmit in domestic poultry, three quail or chickens were infected with 5 × 10^6^EID_50 _of the RGMal/01. At 24 hpi, one additional uninfected bird was introduced into each of the cages housing the infected quail or chickens. The RGMal/01 virus established a respiratory infection in quail without overt signs of disease. Transmission to direct contact quail occurred readily at 3 dpi consistent with previous observations [24] (Table 3). These results are highly significant considering the fact that H7 viruses have been frequently found in live bird markets in the United States in which quail are a common item [25]. In contrast, the RGMal/01 virus replicated less efficiently in white leghorn chickens and was not transmit to direct contact chickens. RGMal/01 virus was absent in the lungs of inoculated or contact chickens at 3 dpi, whereas virus was readily detected in quail (data not shown), consistent with the replication and transmission studies.

**Table 3:** Replication and transmission study of RGMal/01 in chickens and quail^a^.

Groups	Number with positive tracheal swab/total N (Log_10_EID50/ml ± SD)					
	
	Day 1	Day 3	Day 5	Day 7	Day 9	Day 11
Inoculated quail^b^	3/3	3/3	3/3 (4.6 ± 0.6)	3/3	3/3	0/3
Contact quail^b^	0/3	3/3	3/3	3/3	3/3	1/3
Inoculated chicken^b^	3/3	2/3 (2.7 ± 0)	0/3	0/3	0/3	0/3
Contact chicken^b^	0/3	0/3	0/3	0/3	0/3	0/3

^a ^Groups of three birds were inoculated orally, intraocularly, intranasally, and intratracheally with 5 × 10^6 ^EID_50 _of RGMal/01/ml. A volume of 0.6 ml or 1.0 ml of virus inoculum was used for quail and chickens, respectively. The next day after infection, three naïve birds were introduced into the same cage as the infected birds. Tracheal and cloacal swab samples were collected from the chickens every 2 days for 11 days after inoculation

^b ^Cloacal swabs are negative for virus isolation.

### Replication of the RGMal/01 virus in mice

**Figure 2:**
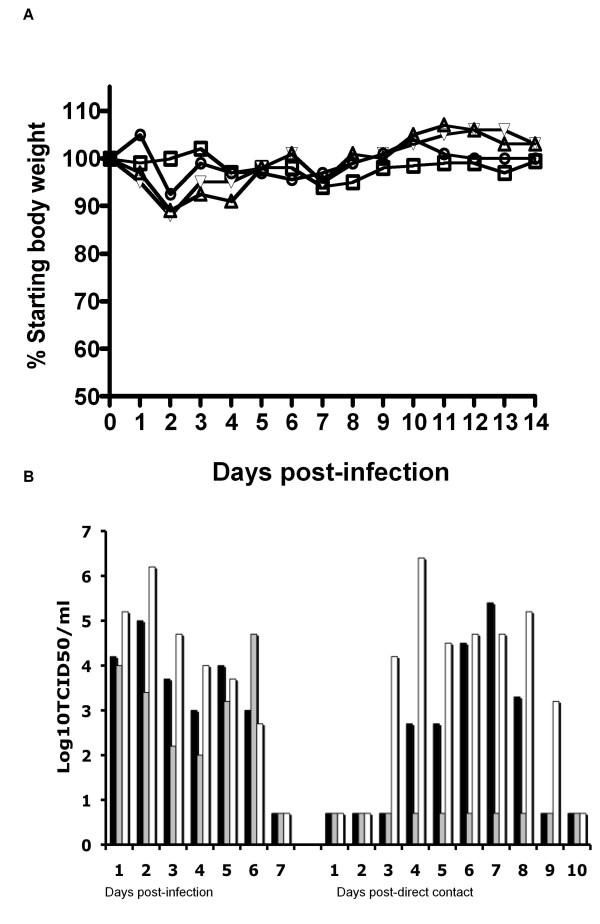
**Replication of the RG Mal/01 in mice and ferrets**. (A) Four five-week-old BALB/c mice were infected under isoflurane anesthesia with 5 × 10^5^TCID_50_/50 μl of RGMal/01. Body weight was measured for 14 days after infection. Body weight was compared with the body weight on day "0" before infection. (B) Three ferrets were inoculated i.n. with 5 × 10^5^TCID_50 _of RGMal/01. Twenty-four hours later, one naïve ferret was added to the same cage as each of the infected ferrets. Viral titers were measured in nasal washes collected daily and were titrated in MDCK cells. The titers are expressed as log_10 _numbers of TCID_50_/ml. The detection limit is 0.699 log_10_TCID_50_/ml.

In order to determine the ability of the RGMal/01 virus to replicate in mammals, we inoculated mice by the i.n. route with 5.0 × 10^1 ^to 5 × 10^5^TCID_50 _of the RGMal/01 (3 mice/dose). Mice infected with 500 TCID_50 _or more had reached 4 to 6 log_10_TCID_50_/lung with escalating virus doses (Table 4). No virus was detected in the brain of mice regardless of the inoculation dose used (data not shown). In an independent study, 4 mice infected with 5 × 10^5 ^TCID_50 _of the RGMal/01 were monitored and weighed daily for 14 days in order to determine the clinical response to infection. Slight body weight loss was recorded between days 2 and 3 pi, but mice showed no clinical signs of disease throughout the observation period (Fig. 2A). Taken together, these studies indicate that the RGMal/01 virus can replicate in mice without previous adaptation. They also underscore the need to better understand the ability of these viruses to infect mammals sub clinically and generate strains with potentially novel features.

**Table 4:** Infectivity of the RGMal/01 virus in mice^a^

Virus infection dose (TCID50)	No. with positive titer in lung/total no. (Log_10_TCID_50_/lung ± SD)
5 × 10^5^	3/3 (5.6 ± 0.4)
5 × 10^4^	3/3 (5.2 ± 0.3)
5 × 10^3^	3/3 (4.5 ± 0.4)
500	3/3 (3.7 ± 0.6)
50	0/3

^a ^4–5 week old female BALB/c mice were anesthetized by isoflurane and infected i.n. with the RGMal/01 virus at a dose 50, 500, 5.0 × 10^3^, 5.0 × 10^4^, 5 × 10^5 ^TCID_50_/50 μl. At day 3 post-inoculation, lungs from three infected mice were collected and homogenized to titrate the virus by TCID_50 _assay in MDCK cells.

### Replication and transmission of the RGMal/01 virus in ferrets

We used the ferret model to further characterize the replication and transmission of RGMal/01 in mammals. Three ferrets housed in separate cages were inoculated i.n. with 5 × 10^5 ^TCID_50 _of the RGMal/01 virus. At 24 hpi, one naïve ferret was introduced into the same cage in direct contact with the infected ferret. In addition, respiratory droplet contact ferrets were included as previously described [26]. Signs of disease, changes in body temperature and body weight, as well as virus shedding were monitored daily. No signs of overt disease, such as lethargy, anorexia, sneezing and cough, were observed in any of the inoculated animals. Transient elevation of body temperature (1.1–1.6°C) was detected in the 3 infected ferrets at day 2 pi (Table 5). Maximum body weight loss in the infected ferrets was less than 4%. Virus was detected in all the inoculated ferrets, as demonstrated by the detection of virus in nasal washes using Flu DETECT™ Antigen Capture Test Strip (not shown), and viral titration, with peak titers ranging from 10^4.7 ^to 10^6.2 ^TCID_50_/ml (Fig 2B). All of the infected ferrets cleared the viruses by 7 dpi. The virus transmitted to two out of three direct contact ferrets at 3 or 4 dpi, and peak titers reached between 10^5.4 ^to 10^6.4 ^TCID_50_/ml, respectively. The contact ferret that shed greatest virus quantities (10^6.4 ^TCID_50_/ml) shared the cage with the inoculated ferret that also shed the highest peak virus titers (10^6.2 ^TCID_50_/ml). Anti-Mal/01 HI antibodies were detected in all three inoculated ferrets as well as in the two direct contact ferrets that shed virus, but not in the contact ferret that did not shed virus in nasal secretions (Table 5). Therefore our data indicate that the RGMal/01 virus replicated efficiently in ferrets and was transmissible to direct contact ferrets. None of the three respiratory droplet contacts were positive for virus isolation or seroconversion (data not shown), indicating that the RGMal/01 was not able to transmit via aerosolized respiratory droplets. This finding is consistent with the lack of efficient transmission of this virus subtype among humans.

**Table 5:** Clinical signs and seroconversion in ferrets infected with RGMal/01.

Animals	Virus detected in nasal wash^a^	Max body temperature rise (°C)	Max body weight loss (%)	Lethargy (Day of onset)	Sneezing (Day of onset)	Serum (HI titer)^b^
Inoculated	3/3	1.1,1.1,1.6	3.9, 3.8, 2.8	0/3	0/3	80, 80,160
Direct Contact	2/3	0.9, 0, 1.7	0, 1.0, 9.3	0/3	0/3	<10, 80,160

^a ^Virus in nasal washes was analyzed using Flu DETECT ™ Antigen Capture test Strip (Synbiotics Corp.) and titrated by TCID_50 _assay in MDCK cells

^b^Blood was collected at 14 dpi, RGMal/01 was used in the HI assay to detect anti-H7 antibodies.

**Figure 3:**
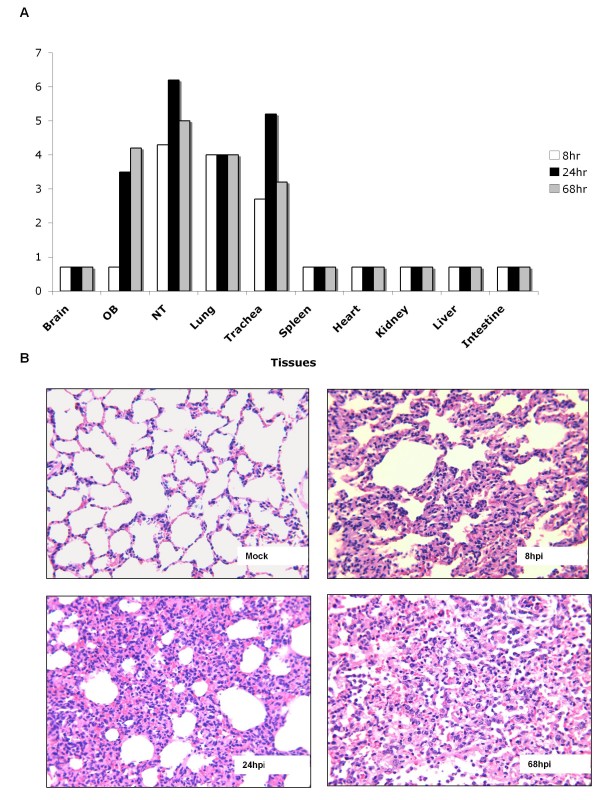
**Replication kinetics and histopathology of the RGMal/01 in inoculated ferrets**. Three ferrets were inoculated i.n. with 5 × 10^5^TCID_50 _of RGMal/01 virus. At 8, 24, and 68 hpi, each ferret was euthanized. (A) Brain, olfactory bulb (OB), nasal turbinate (NT), trachea, lung, kidney, liver, spleen and intestine were harvested, weighted and homogenized in PBS. Virus in these tissues was titrated in MDCK cells. The titers are expressed as log_10 _numbers of TCID_50 _per ml of 10% (w/v) tissue homogenate. The detection limit is 0.699 log_10_TCID_50_/ml. (B) Lung was collected and fixed with formalin. The lung of a mock-infected ferret was collected as negative control. Sections of 5 μm thick were cut and routinely processed for H&E staining. Note that the severe inflammatory infiltration is progressing in the infected lung.

To evaluate the virus replication kinetics and tissue tropism in the inoculated ferrets, we infected 3 ferrets i.n. with 5 × 10^5 ^TCID_50_of the RGMal/01 and collected samples from different tissues at 8, 24 and 68 hpi. As shown in Fig 3A, high levels of virus replication were detected in upper (nasal turbinate and trachea) and lower respiratory tract (lung). The virus showed peak titers of 10^6.2 ^TCID_50 _in nasal turbinate and trachea 10^5.2 ^TCID_50 _at 24 hpi, then declined by 68 hpi, whereas virus titers in lungs were maintained at a similar level at all 3 time points. The virus did not spread to the brain but it was detected in the olfactory bulbs. No virus was isolated from the spleen, kidney, heart, liver and intestine at any of the time points. Histological examination of the lung of inoculated ferrets revealed moderate interstitial pneumonia and evident infiltration of inflammatory cells, including mononuclear cells, lymphocytes and neutrophils, noticeable by 24 hpi and becoming more severe by 68 hpi (Fig. 3B). No evident lesions were observed in tracheas from the inoculated ferrets at any time point during infection (data not shown).

## Conclusion

Avian influenza viruses of H7 subtypes are frequently isolated from domestic poultry and have recently become of a significant public health concern due to their propensity to transmit directly from infected poultry to humans. Previous studies with HPAI H7 and LPAI H7 isolated from domestic land-based birds have shown that these viruses can replicate in mice and ferrets without adaptation [16-18,27]. Aquatic birds are the natural reservoir for influenza A viruses, however little is known about the potential of viruses from this primordial reservoir to infect mammals. In this report we performed biological and molecular characterizations of a H7N3 virus isolated from a mallard in Canada, 2001. Our results indicated that 7 of the gene segments of the Mal/01 share high level of similarity with influenza A/Canada/rv504/2004 (H7N3), the HPAI virus isolated from a human conjunctivitis case in Canada in 2004. Interestingly, the RGMal/01 virus replicated well in HAE cells despite its strict SAα2–3Gal receptor-binding specificity. Consistent with this observation, the virus was able to infect mice and replicate and transmit in ferrets without causing substantial morbidity and with no mortality.

An outbreak of avian influenza in 2004 in British Columbia, Canada, included LPAI and HPAI viruses of the H7N3 subtype. Our genomic sequence analysis of Mal/01 indicated that with the exception of NS, all other genes encoded proteins that share more than 97% amino acid sequence similarity with influenza A/Canada/rv504/2004 (H7N3) from a human case linked to the poultry outbreak. Thus, it is tempting to speculate that a virus with the potential to infect mammals was circulating in wild birds in Canada as early as in 2001. The H7N3 mallard virus did not replicate particularly well in chickens. However, it was able to replicate and transmit readily in Japanese quail, a common item in live bird markets in which H7 viruses have been frequently found [28-30].

RGMal/01 replicated to high titers in the respiratory tracts of mice and ferrets. However, efficient replication of a LPAI in the mammalian respiratory tract is not completely unprecedented. In an earlier study, we found that the HA of recent H9N2 viruses (including RGWF10) that contain leucine (L) at position 226 of the HA receptor-binding site (H3 numbering) bind efficiently to α2–6-linked SA moieties. The virus with L226 replicated more efficiently in ferrets than the virus with glutamine at this position and it was able to transmit to contact ferrets [19,31]. Interestingly, in the present study we observed that the RGMal/01 virus, which has typical avian receptor binding preference for α2–3-linked SA, was present in nasal secretions at higher concentrations than another avian virus, WF10. Nevertheless, RGMal/01 was shed at lower concentrations than a prototype H3N2 human influenza virus, Mem/98 [31]. High levels of RGMal/01 virus shedding were detected from both the upper and the lower respiratory tract of ferrets. Similarly, Belser et al. observed that, without causing substantial morbidity or mortality, some North American H7 viruses, isolated from humans or poultry, replicated efficiently in the respiratory tract of both mice and ferrets [18]. Virus titers measured in the respiratory tract of the ferrets were even higher than those observed following infection with human H3N2 viruses [18]. In addition, high titers of virus (ranging from 10^6.3 ^to 10^7.8 ^EID50/ml) were recovered in nasal washes from ferrets inoculated with some of the H5N1 viruses having high binding affinity to SAα2–3Gal receptor [32]. Therefore, our data are consistent with other findings and suggest that LPAI avian influenza viruses do not require high binding affinity to human-like receptors in order to replicate efficiently in the upper respiratory tract of ferrets. More importantly, our results are consistent with the notion high levels of nasal shedding are necessary but not sufficient to achieve efficient contact (or droplet) transmission of influenza viruses in ferrets.

Although the mallard H7N3 virus has nearly exclusive SAα2–3Gal receptor binding specificity and replicates predominantly in ciliated HAE cells, it reaches titers similar to another avian virus with human-like receptor binding specificity which replicates in non-ciliated cells. Considering that the RGMal/01 virus antigen co-localized only with ciliated cells after multiple replication cycles in HAE cells, it is unlikely that other cell types served as substrate for virus multiplication. The efficient replication in the SAα2–3Gal receptor-containing cells might provide a possible explanation for efficient replication of the RGMal/01 in the upper respiratory tract of ferrets: the virus is predicted to replicate efficiently in ciliated airway epithelial cells populations. Similarly, productive H5N1 viral replication can still be detected in *ex vivo* cultures of human nasopharyngeal, adenoid, and tonsillar tissue when infected with high MOI of 5.0 [33].

To our knowledge, this is the first report showing that an avian influenza H7 virus isolated from aquatic birds replicates in and is transmitted to ferrets by direct contact without prior adaptation by serial passage. Our results in HAE and ferret models are consistent with the limited human-to-human transmission following humans' infections with H7 viruses with typical avian-like characteristics [34]. Belser et al previously reported similar findings with H7N2 viruses isolated from poultry. However, findings with H7 viruses contrast transmission studies of H5N1 viruses in ferrets [32,35]. Although partial transmission of recent H5N1 strains to contact ferrets was detected by seroconversion, usually little or no virus was present in the nasal washes of contact ferrets [32,35].

Additional functional changes would seem to be required to enable the RGMal/01 virus for sustained aerosolized respiratory droplet transmission, which might include, among other features, switching to α2–6-sialoglycan binding preference. A recent study indicated that affinity for human-like α2–6-linked SA receptors may not be sufficient to allow efficient transmission of H5N1 virus among ferrets [35], suggesting that additional structural features of the receptor or other yet unidentified viral functions are critical as well. For example, the topology of long chain α2–6-sialylated glycan receptors on the human upper respiratory tissues may play such role [36]. An avian/human H9N2 reassortant virus, which already displayed human-like receptor specificity, required adaptation by serial passage in ferrets in order to obtain a strain that was transmitted by respiratory droplets [26]. It remains to be elucidated whether a similar approach would lead to respiratory droplet transmission of a H7 subtype virus. Consequently, it is also essential that avian surveillance programs contemplate the analysis of receptor specificity of strains isolated from the field in the context of other *in vitro *and *in vivo *approaches to clearly delineate their zoonotic potential.

At the time of writing this report, an influenza pandemic was declared, caused by subtype H1N1 triple reassortant virus of swine origin containing genes derived from human, avian and swine influenza viruses. Noteworthy is the fact that this new reassortant lacks most of the features that scientists have come to recognize as virulence markers of influenza viruses in humans. It does not encode the virulence marker PB1F2, the NS1 is 219 amino acids long and thus lacks the PDZ domain, PB2 does not encode lysine 627, among other features. In this regard, it is important to note that the NS gene of Mal/01 belongs to allele B, which to our knowledge has not been associated with infections in mammals. However, this gene does not appear to have been an impediment for the replication of the RGMal/01 virus in mice or for the efficient replication and partial transmission in ferrets. Thus, further studies are needed in order to better identify and characterize viral determinants of virulence and transmission of avian influenza viruses in mammals.

## Materials and methods

### Viruses and cells

The A/Mallard/Alberta/24/01 (H7N3) (Mal/01), A/Guinea fowl/Hong Kong/WF10/99 (H9N2) (WF10) and A/Panama/2007/99 (H3N2) (Pan/99) viruses were obtained from the influenza repository at St. Jude Children's Research Hospital, Memphis, TN. Avian viruses were propagated in 10-day-old embryonated, specific-pathogen-free (SPF) chicken eggs, while the human influenza virus was propagated in Madin-Darby canine kidney (MDCK) cells. Virus stocks were maintained at -80°C until use. MDCK cells were maintained in modified Eagle's medium (MEM) (Sigma-Aldrich, St. Louis, MO) containing 5% fetal bovine serum (Sigma-Aldrich, St. Louis, MO). 293-T human embryonic kidney (HEK) cells were cultured in OptiMEM I (GIBCO, Grand Island, NY) containing 5% FBS. Passage 1 human airway epithelial cells (HAE) were purchased from Cell Applications, Inc. (San Diego, CA). The cells were expanded and differentiated as described previously [19]. The median tissue culture infectious dose (TCID_50_) of each virus was determined using MDCK cells. Avian influenza viruses were also titrated by the Reed and Muench [37] method to determine the 50% egg infectious dose (EID_50_).

### Viral gene cloning and sequencing

Total viral RNA was extracted from infected allantoic fluid using the RNeasy kit (Qiagen, Valencia, CA, USA) in accordance with manufacturer's instructions. Reverse transcription was carried out with the uni12 primer (5'-AGCAAAAGCAAGG-3') and AMV reverse transcriptase (Promega, Madison, WI, USA) [38]. PCR amplification was performed using universal primers described by Hoffmann et al [38] as well as specific primers (primer sequences available upon request). PCR products were sequenced using the BigDye-Terminator protocol V3.1 (Applied Biosystems, Foster City, CA). For the generation of H7N3 virus by reverse genetics, PCR products corresponding to each of the 8 genes were purified and cloned into the vector pDP2002. Plasmid sequences were compared to the sequences generated from the wild type virus (obtained by direct sequencing of the RT-PCR products). Only clones that exactly matched the parental virus sequence were used for virus rescue by reverse genetics. Reverse genetic Mal/01 (RGMal/01) was rescued according to the protocol described previously [39]. The nucleotide sequences determined in this study are available from GenBank (Table 1). The reverse genetics-generated WF10 (RGWF10) virus has been previously described [29].

### Sialoglycan microarray analysis of whole virions

RGMal/01 virus was propagated in 10-day-old embryonated chicken eggs as described previously [19]. Virus infectivity was inactivated by 0.02% β-propiolactone treatment (Sigma-Aldrich, St. Louis, MO). Allantoic fluid containing inactivated virus was clarified by low-speed centrifugation (1,000 *g*, 5 minutes) and concentrated by using centrifugal filters (Centricon Plus-70^® ^Millipore, Billerica, MA). Virus was adjusted to a final concentration of 128 chicken erythrocyte hemagglutination units per 50 μl in PBS containing 3% bovine serum albumin (BSA). Glycan microarray binding analysis was performed as previously described [20,31]. Briefly, virus bound on the arrays was detected with ferret anti-H7 antibody or control serum in PBS-BSA followed by biotinylated anti-ferret IgG, respectively. Bound antibody complexes were detected with Alexa Flour 488 labeled streptavidin. Mock-infected allantoic fluid was used as negative control (not shown).

### Growth curve in HAE cells

Growth curves in HAE cells were performed as described previously [19]. Briefly, duplicate HAE cultures growing in 12 mm-diameter inserts were inoculated with each virus via the apical side at an MOI of 0.2. After incubation at 35°C for 1 h, the inoculum was removed, the cells were washed five times with 200 μl of growth medium and subsequently incubated at 37°C in 5% CO2. At different time points post-inoculation, 200 μl of growth medium was added to each culture to harvest, allowed to mix for 10 min at 37°C and an equal volume of culture medium containing progeny viruses was harvested, aliquoted, and stored at -80°C. Virus TCID50 titers were determined in MDCK cells.

### Double immunostaining to determine infection of different HAE cell types

Infected HAE cultures were thoroughly washed with growth medium, fixed with 4% paraformaldehyde, and then permeabilized with 0.2% Triton X-100. Potential endogenous peroxidase activity was eliminated with 1% H_2_O_2_-methanol. After being blocked with 1% BSA-PBS, the cells were incubated with a specific monoclonal antibody against β-tubulin (Sigma, St. Louis, MO), the cellular marker of ciliated cells, followed by incubation with peroxidase-conjugated goat anti-mouse immunoglobulin G (IgG; Sigma, St. Louis, MO). The cilia were visualized by developing the cells in Vector SG substrate (Vector Laboratories, Inc., Burlingame, CA). After being washed with PBS and blocked with 1% BSA in PBS, the virus-infected cells were incubated with chicken antisera against avian influenza viruses prepared in our laboratory (with HA inhibition titers of >320), followed by incubation with peroxidase-conjugated goat anti-chicken IgG (Kirkegaard & Perry Laboratories, Gaithersburg, MD). The viral antigen was visualized by incubating the cells in AEC solution (Sigma, St. Louis, MO). The cultures were mounted and en face to be photographed for representative images. To determine the number of infected cells and the tropisms of the viruses, the stained cultures were observed at 400× magnification.

### Replication and transmission studies in birds, mice, and ferrets

Four to six-week old Japanese quail (*Coturnix coturnix*, University of Maryland, College Park, Central Animal Research Facility) and three to four-week White Leghorn chickens (Charles River Laboratories, Wilmington, MA) were used. Groups of three birds were inoculated via the oral, intraocular, intranasal, and intratracheal routes with 5 × 10^6 ^EID_50 _of RGMal/01 per ml. A volume of 0.6 ml or 1.0 ml of virus inoculum was used for quail and chickens, respectively. Three quail (or chickens) were introduced at 1 day post-infection (dpi) into the cage where the infected quail (or chickens) were kept. Water and food bowls as well as cage liners were changed in order to prevent transmission of virus via contaminated feed or water. Tracheal and cloacal swabs were collected at 1, 3, 5, 7, 9, 11 dpi and stored in glass vials in 1 ml of freezing medium [50% glycerol in phosphate saline buffer (PBS) – containing antibiotics] and titrated in 10-day embryonated chicken eggs by the method of Reed and Muench [37]. Birds were observed and scored daily for clinical signs of illness.

BALB/c mice (4–5 week old females, Charles River) were anesthetized and infected intranasally (i.n.) with the RGMal/01 virus at a dose of 50, 500, 5.0 × 10^3^, 5.0 × 10^4^, 5 × 10^5 ^TCID_50_/50 μl. One group of four mice was monitored and weighed daily for 14 days, whereas a second group of three mice was sacrificed at 3 dpi, brains and lungs were collected and homogenated to titrate the virus by TCID50 in MDCK cells.

Female Fitch ferrets, 3 to 6 months old (Triple F farms, Sayre, PA), were used to test virus replication and transmission. Prior to infection, ferrets were monitored for 3–5 days to measure body weight and establish baseline body temperatures. Temperature readings were recorded daily through a transponder (Bio Medic data systems, Seaford, DE) implanted subcutaneously in each ferret. Blood was collected at 3 days before infection, and serum was tested for antibodies against RGMal/01 virus using the hemagglutination inhibition (HI) assay. Ferrets with HI titers at or lower than 10 were considered "influenza A-free" and were used in the study. Ferrets were housed in wire cages placed inside Hepa-filter isolators. Each ferret group housed in a single isolator unit consisted of three ferrets: one infected, one direct contact and one aerosol contact, as described previously [26,31]. Three groups (9 ferrets) were used in this study. In each group, one ferret was lightly anesthetized with ketamine (20 mg/kg) and xylazine (1 mg/kg) via intramuscular injection and inoculated i.n. with 5 × 10^5^TCID_50 _of RGMal/01 in PBS, 250 μl per nostril. Twenty-four hours later, two naïve ferrets were introduced. One (direct contact) was introduced directly into the cage with the infected ferret while the other (aerosol contact) was placed in a cage separated from the infected ferrets by a wire mesh (1 mm openings). The wire mesh prevented physical contact between the aerosol and the infected and direct contacts, allowing only air to be shared between the ferrets. All materials inside the cage of inoculated ferrets were removed and replaced before introducing the direct contact to preclude fomite transmission. Temperatures and body weight readings were taken daily. To monitor viral shedding, nasal washes were collected daily for up to 14 days. Briefly, ferrets were anesthetized as described above, and 1 ml of PBS was used to induce sneezing. Nasal washes were collected into petri dishes and brought to a total volume of 1 ml with PBS. Nasal washes were immediately tested for virus using the Flu DETECT™ Antigen Capture Test Strip (Synbiotics Corp., San Diego, CA) and additional aliquots were stored at -80°C before performing TCID_50 _assays. Two weeks after infection, blood was collected and seroconversion was monitored by determining HI antibody titers.

To study the kinetics of virus replication and tissue tropism in ferrets, 3 ferrets were inoculated i.n. with 5 × 105 TCID50 of RGMal/01 as described above. At 8, 24, and 68 hours post-infection (hpi), each ferret was euthanized. Nasal turbinates, lungs, kidneys, olfactory bulbs (OB), brains, livers, spleens and intestines were harvested, and samples were both fixed with buffered neutral formalin for histological evaluation and stored at -80°C for virus titration. For histopathology, paraffin-embedded sections of 5 μm thicknesses were cut and stained with hematoxylin and eosin (H&E) (Histoserv, Inc., Germantown, MD). Representative microscopic photos were taken with SPOT ADVANCED software (Version 4.0.8, Diagnostic Instruments, Inc., Sterling Heights, MI). The virus titer for each organ was determined by the method described by Reed and Muench and was expressed as log_10 _numbers of TCID_50 _per ml of 10% (w/v) tissue homogenate.

Animal experiments were carried out under BSL-2 (birds and mice study) or BSL-2+ (ferret study) conditions and conducted under guidelines approved by the IACUC of the University of Maryland.

## Competing interests

The authors declare that they have no competing interests.

## Authors' contributions

HS generated the reverse genetics version of the Mal/01 virus, performed in vitro and in vivo studies, and wrote the manuscript. HW performed in vitro studies and wrote the manuscript. YA performed in vivo studies. DRP developed the conceptual aspects of the work and wrote and edited the manuscript. All authors read and approved the final manuscript.
